# Causal Inference Between Chronic Periodontitis and Chronic Kidney Disease: A Bidirectional Mendelian Randomization Analysis in a European Population

**DOI:** 10.3389/fgene.2021.676136

**Published:** 2021-06-07

**Authors:** Jie Yang, Tianyi Chen, Yahong Zhu, Mingxia Bai, Xingang Li

**Affiliations:** ^1^Division of Nephrology, Beijing Jishuitan Hospital, Beijing, China; ^2^Beijing Lucidus Bioinformation Technologies, Beijing, China; ^3^Department of Stomatology, Beijing Jishuitan Hospital, Beijing, China; ^4^Centre for Precision Health, Edith Cowan University, Joondalup, WA, Australia; ^5^School of Medical and Health Sciences, Edith Cowan University, Joondalup, WA, Australia

**Keywords:** causal inference, chronic periodontitis, chronic kidney disease, Mendelian randomization, single nucleotide polymorphisms

## Abstract

**Background:**

Previous epidemiological studies have shown significant associations between chronic periodontitis (CP) and chronic kidney disease (CKD), but the causal relationship remains uncertain. Aiming to examine the causal relationship between these two diseases, we conducted a bidirectional two-sample Mendelian randomization (MR) analysis with multiple MR methods.

**Methods:**

For the casual effect of CP on CKD, we selected seven single-nucleotide polymorphisms (SNPs) specific to CP as genetic instrumental variables from the genome-wide association studies (GWAS) in the GLIDE Consortium. The summary statistics of complementary kidney function measures, i.e., estimated glomerular filtration rate (eGFR) and blood urea nitrogen (BUN), were derived from the GWAS in the CKDGen Consortium. For the reversed causal inference, six SNPs associated with eGFR and nine with BUN from the CKDGen Consortium were included and the summary statistics were extracted from the CLIDE Consortium.

**Results:**

No significant causal association between genetically determined CP and eGFR or BUN was found (all *p* > 0.05). Based on the conventional inverse variance-weighted method, one of seven instrumental variables supported genetically predicted CP being associated with a higher risk of eGFR (estimate = 0.019, 95% CI: 0.012–0.026, *p* < 0.001).

**Conclusion:**

Evidence from our bidirectional causal inference does not support a causal relation between CP and CKD risk and therefore suggests that associations reported by previous observational studies may represent confounding.

## Introduction

Chronic kidney disease (CKD) leading to end-stage renal disease (ESRD) and requiring dialysis or kidney transplantation is greatly associated with shortened life expectancy (Webster et al., 2017). The prevalence of CKD is around 10% worldwide, which is becoming one of the major burdens for health care in every country (GBD 2017 Risk Factor Collaborators, 2018). CKD demonstrates a series of changes in glomerular, tubular, and endocrine renal structures. As the pathogenesis of CKD is considerably complicated and yet to be uncovered, the current therapeutic options for CKD are limited to controlling its risk factors, such as blood pressure, diabetes, and chronic inflammation (Köttgen et al., 2010).

As a low-grade chronic inflammation, chronic periodontitis (CP) has been reported to be highly associated with kidney function measures, including estimated glomerular filtration rate (eGFR) and blood urea nitrogen (BUN) (Kinane et al., 2017). Meanwhile, greater deterioration of periodontal status, including poor oral hygiene and gingival, has been observed among CKD patients, especially those under dialysis treatment, than health controls (Gautam et al., 2014; Tadakamadla et al., 2014). Due to the restriction of methodological bias, it is a particular challenge to determine the causality by conventional observational study, in terms of the existing of confounding, reverse causation, and measurement error (Boyko, 2013). Therefore, investigating the causal relationships between CP and CKD through other effective approaches is of great urgency for disease prevention and treatment strategies (Nanayakkara and Zhou, 2019).

Mendelian randomization (MR) is a powerful genetic epidemiological tool used to evaluate causal effects, overcoming the limitations of conventionally observational studies (Smith and Ebrahim, 2003). Two-sample MR analysis is an extensive application of the MR approach, allowing the use of GWAS summary statistics for MR studies rather than limiting them using individual-level data within one sample (Burgess et al., 2015). Using two-sample MR analysis, the causal relationship between CP and risk factors of CKD, e.g., cardiovascular disease (Bell et al., 2020) and blood pressure (Yu et al., 2020), has been assessed. In this study, we took advantage of the recent large-scale meta-analysis of the GWAS of CP and CKD to bidirectionally perform a two-sample MR analysis for examining the causal associations between these two diseases.

## Materials and Methods

### Study Design

In this bidirectional two-sample MR analysis, genetic variants were used to investigate the causal effect and direction of CP with eGFR (the primary kidney function trait) and BUN (the second kidney function trait). Briefly, the modifiable risk factor-associated single-nucleotide polymorphisms (SNPs) hired as instrumental variables (IVs) are randomly allocated obeying Mendel’s law of independent assortment. The SNPs are distributed at the forming of the zygote, which always precedes the onset of disease and is less likely to be affected by confounding or reverse causation. Therefore, grouped by the naturally allocated genetic IVs, the MR approach mimics a randomized controlled trial using individual- or summary-level data from observational studies (Smith and Hemani, 2014).

**FIGURE 1:**
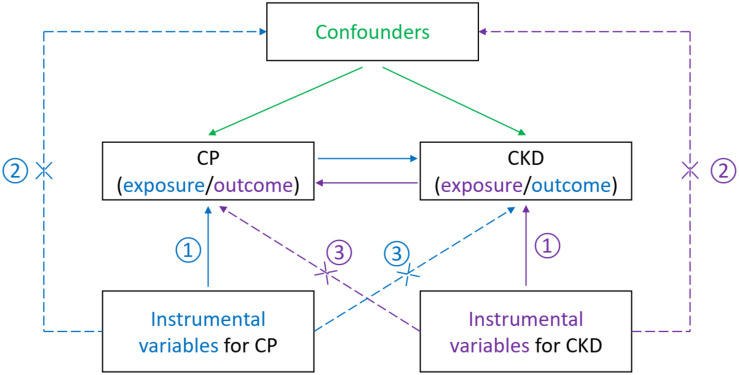
Illustration of the current bidirectional Mendelian randomization setting with required instrumental variable assumptions. That is, each genetic variant (SNP) is ① associated with the exposure (a disease or phenotypic characteristics), ② but not associated with unmeasured confounders of the exposure–outcome association, and ③ not associated with the risk of outcome (another disease or phenotypic characteristics) conditional on the exposure and confounder. The blue items indicate the assumptions for causal inference of CP on CKD, while the purple items show the assumptions for reverse inference. The green items present confounders of the exposure–outcome association. CP, chronic periodontitis; CKD, chronic kidney disease; SNP, single nucleotide polymorphism.

To obtain reliable results, the valid IVs must satisfy three important assumptions within the MR analysis process (Smith et al., 2020): (1) the IVs are solidly related to the exposure, (2) the IVs are not correlated with any confounders influencing both exposure and outcome, and (3) the IVs affect the outcome only through their effects on the exposure and not through any other causal pathways (Figure 1). Details on the MR design have been described elsewhere (Burgess and Thompson, 2015; Tillmann et al., 2017). For each inference direction, the analysis included three main procedures: the selection of suitable genetic IVs for the corresponding exposure, application of multiple MR methods, and pleiotropic effect analyses, as described below.

### Participants and Data Sources

For our study, summary statistics of the genome-wide association study (GWAS) for CP was derived from the Gene-Lifestyle Interactions in Dental Endpoints (GLIDE) Consortium, analyzing a total of 12,289 clinically diagnosed periodontitis cases and 22,326 controls (Shungin et al., 2019). Summary-level data of CKD concerning kidney function measures (i.e., eGFR and BUN) were extracted in currently the biggest the Chronic Kidney Disease Genetics (CKDGen) Consortium (including 41,395 cases and 4,39,303 controls) (Wuttke et al., 2019).

The participants of these two GWAS studies are mostly people with European ancestry. In both these corresponding original studies, all participants provided written informed consent. Each study included in the GLIDE and the CKDGen Consortiums was approved by a local institutional review board and an ethics committee.

### Selection of Genetic Instrumental Variables

All genetic instrumental variables for the current bidirectional MR analysis were filtered to fit the three basic MR assumptions as described above.

For the first assumption, the genetic instruments for estimating the causal effect of CP on the risk of CKD were obtained from a GWAS analysis of the GLIDE Consortium, in which eight SNPs were suggestively (*p* < 5 × 10^–6^) associated with periodontitis (Shungin et al., 2019). To investigate the causal effect of kidney function on chronic periodontitis, 256 and 75 index SNPs reported significantly associated with eGFR and BUN in a meta-analysis of GWAS on kidney function (*p* < 5 × 10^–8^) were included as candidate genetic instruments, respectively.

The assumptions of genetic instrumental variables being independent of outcome and confounding factors were investigated for the genome-wide significant associations with corresponding outcome variables and their related confounding factors by searching in a web-based GWAS catalog^1^ (Staley et al., 2016). The SNPs co-associated with outcome variable and potential confounders were removed to satisfy these two assumptions (Smith et al., 2008).

Besides, pairwise linkage disequilibrium clumping (Purcell et al., 2007) was performed for identifying the independent signals among correlated SNPs, and the removal of correlated SNPs was conducted by Steiger filtering for the exclusion of reverse causality (Hemani et al., 2017).

### Statistical Analysis

We employed multiple complementary methods of MR for a comprehensive and precise causal effect investigation, including the inverse variance weighted (IVW) (Burgess and Thompson, 2015), the weighted median (WM) (Bowden et al., 2016), and the Mendelian randomization–Egger (MR-Egger) (Burgess and Thompson, 2017) methods. For the fundamental estimates of the causal effect of the exposure on the risk of the outcome, we performed the IVW method which is conventionally used in two-sample MR studies (Burgess and Thompson, 2015). The IVW method uses the associations (beta-coefficients and standard errors) combined with risk factors and the results of regressing each genetic variant in turn, using summarized data from all the genetics variants to estimate causality (Rees et al., 2019). The WM method could give consistency analyses by calculating a single weighted median estimator for combining data on multiple genetic instruments (Bowden et al., 2016). Compared to the IVW method which only provides consistent results when all genetic variants in the analysis are valid IVs, the WM method could give a consistent estimator even if some of the genetic instruments in the reference are not valid instrument variables (Hartwig et al., 2017). MR–Egger methods provide assess potential asymmetry for bias from the pleiotropic effect of the multiple genetic variants and give estimates of the causal effect (Verbanck et al., 2018). MR–Egger has the advantages to assess the directional pleiotropy under the weaker assumption (Burgess and Thompson, 2017), e.g., the Instrument Strength Independent of Direct Effect (InSIDE) assumption.

The power analysis was conducted by a web-based application^2^ (Brion et al., 2013) to evaluate the minimum detectable magnitude of association for outcomes in bidirectional causal inferences between CP and CKD. All results are presented as an estimate or odds ratio (OR) with a 95% confidence interval (CI) of the outcomes OR or per predicted increase/decrease. All statistical tests were two-sided, and the evidence of association was cutoff at a prespecified *p*-value below 0.05. All analyses were performed in R version 4.0.3 (R Project for Statistical Computing, Vienna, Austria), with packages *MendelianRandomization* (0.5.0) (Yavorska and Burgess, 2017) and *forestplot* (1.10)^3^.

## Results

### Selection of Instrumental Variables for CP and Kidney Function

After the removal of SNPs associated with potential confounders in the online GWAS database, the pairwise linkage disequilibrium clumping and matching of coding alleles between the summary statistics of the exposure and those of the outcome, and the exclusion of correlated SNPs by Steiger filtering, the valid instrumental variables were selected to fit the three basic MR assumptions above. Seven SNPs suggestively associated with CP were selected as genetic instruments for the MR analysis of CP causally associated with CKD. In the reversed MR analysis, six SNPs and nine SNPs significantly associated with two commentary kidney function measures (eGFR and BUN), respectively, were included. The corresponding summary statistics for these SNPs for MR analyses were retrieved from the reported summary GWAS results of CKD and CP, respectively (Tables 1, 2).

**TABLE 1:** Summary statistics for Mendelian randomization analysis of the potential causal effect of chronic periodontitis on kidney function.

**SNP**	**Chr**	**EA**	**OA**	**EAF**	**Exposure: chronic periodontitis**			**Primary outcome: eGFR**			**Secondary outcome: BUN**		
					**Beta**	**SE**	***p***	**Beta**	**SE**	***p***	**Beta**	**SE**	***p***
rs13005050	2	C	T	0.14	0.1432	0.0310	3.76E-06	0.0009	0.0006	1.03E-01	−0.0027	0.0014	6.29E-02
rs4956201	4	C	A	0.89	0.2406	0.0474	3.89E-07	−0.0006	0.0008	4.42E-01	0.0001	0.0020	9.61E-01
rs6816769	4	C	T	0.89	0.1348	0.0294	4.57E-06	0.0004	0.0006	4.81E-01	0.0006	0.0014	6.53E-01
rs78422482	4	A	G	0.01	0.2425	0.0510	2.02E-06	−0.0006	0.0010	5.15E-01	0.0024	0.0024	3.10E-01
rs73155039	7	A	G	0.99	0.8316	0.1757	2.22E-06	−0.0004	0.0019	8.35E-01	0.0034	0.0052	5.23E-01
rs2976950	8	A	G	0.60	0.0963	0.0195	7.99E-07	0.0018	0.0004	4.52E-07	0.0007	0.0010	4.37E-01
rs151226594	11	G	T	0.01	0.3671	0.0768	1.75E-06	0.0012	0.0014	3.90E-01	0.0030	0.0034	3.67E-01

*These SNPs are associated with chronic periodontitis at the genome-wide significance level (p < 5 × 10^–6^) in a meta-analysis with up to 34,615 individuals of European ancestry (Shungin et al., 2019). SNP, single nucleotide polymorphism id; Chr, chromosome; EA, effect allele; OA, other allele; EAF, effect allele frequency; Beta, SNP effect size; SE, standard error; p, p-value; eGFR, estimated glomerular filtration rate; BUN, blood urea nitrogen.*

**TABLE 2:** Summary statistics for Mendelian randomization analysis of the potential causal effect of kidney function on chronic periodontitis.

**SNP**	**Chr**	**EA**	**OA**	**EAF**	**Primary exposure: eGFR**			**Secondary exposure: BUN**			**Outcome: chronic periodontitis**		
					**Beta**	**SE**	***p***	**Beta**	**SE**	***p***	**Beta**	**SE**	***p***
rs11694902	2	A	G	0.76	0.0050	0.0004	3.28E-34				0.0047	0.0257	8.56E-01
rs17462630	2	C	G	0.14	0.0041	0.0005	2.14E-16				0.0026	0.0189	8.91E-01
rs9868185	3	G	A	0.31	0.0055	0.0004	4.04E-37				0.0012	0.0182	9.47E-01
rs12920176	16	A	C	0.79	0.0026	0.0004	1.01E-09				0.0002	0.0186	9.92E-01
rs113445505	19	T	C	0.20	0.0096	0.0005	1.21E-99				−0.0089	0.0184	6.26E-01
rs6127099	20	T	A	0.34	0.0034	0.0004	1.53E-20				0.0001	0.0211	9.94E-01
rs10874312	1	A	G	0.66				0.0070	0.0009	1.73E-14	0.0035	0.0187	8.51E-01
rs760077	1	A	T	0.41				0.0134	0.0010	2.10E-44	−0.0124	0.0184	4.99E-01
rs34773350	2	C	T	0.86				0.0083	0.0012	2.90E-11	0.0034	0.0257	8.94E-01
rs9849724	3	G	T	0.46				0.0047	0.0009	4.09E-08	0.0043	0.0180	8.13E-01
rs4976646	5	C	T	0.34				0.0073	0.0009	2.91E-15	0.0033	0.0196	8.67E-01
rs13230625	7	A	G	0.70				0.0134	0.0013	1.08E-26	0.0077	0.0262	7.71E-01
rs6597862	10	C	A	0.76				0.0058	0.0010	8.33E-09	−0.0011	0.0210	9.59E-01
rs3925584	11	T	C	0.55				0.0096	0.0009	9.85E-29	0.0078	0.0183	6.69E-01
rs4886755	15	G	A	0.51				0.0095	0.0009	1.73E-28	−0.0047	0.0175	7.89E-01

*These SNPs are associated with chronic periodontitis at the genome-wide significance level (p < 5 × 10^–8^) in a meta-analysis with up to 480,698 individuals of European ancestry (Wuttke et al., 2019). SNP, single-nucleotide polymorphism id; Chr, chromosome; EA, effect allele; OA, other allele; EAF, effect allele frequency; Beta, SNP effect size; SE, standard error; p, p-value; eGFR, estimated glomerular filtration rate; BUN, blood urea nitrogen.*

### Causal Association of CP and CKD by Conventional MR Method

**FIGURE 2:**
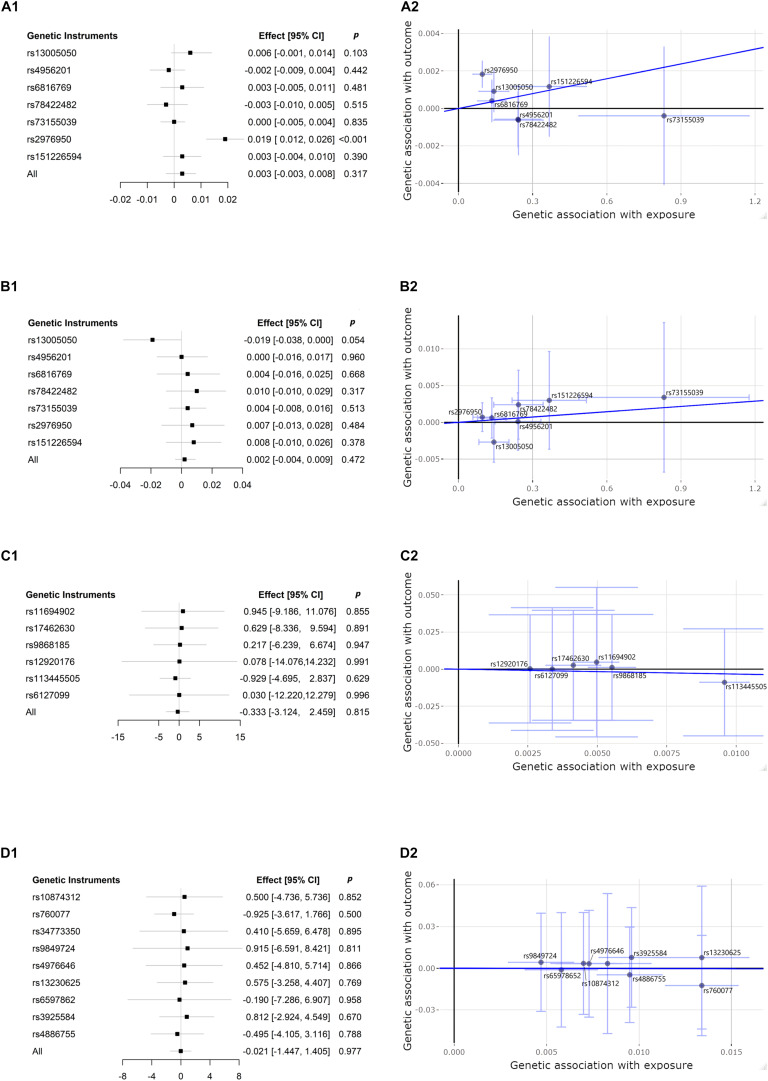
Forest plots and scatter plots of associations between chronic periodontitis (CP) and chronic kidney disease (CKD), dedicated by kidney function measures of estimated glomerular filtration rate (eGFR) and blood urea nitrogen (BUN). CP-related single-nucleotide polymorphism (SNP) and risk of eGFR: **(A1,A2)**; CP-related SNP and risk of BUN: **(B1,B2)**; eGFR-related SNP and risk of CP: **(C1,C2)**; BUN-related SNP and risk of CP: **(D1,D2)**. Forest plots **(A1,B1,C1,D1)** present the estimates with a horizontal line representing 95% confidence intervals (CIs) for the exposure-related SNP allele for outcome risk. Scatter plots **(A2,B2,C2,D2)** present the per-allele association with outcome risk plotted against the per-allele association with one standard deviation of exposure (with vertical and horizontal purple lines showing the 95% CI for each SNP). The slope of the navy solid line in the scatter plots corresponds to each Mendelian randomization (MR) estimate. *p*, *p*-value.

We estimated the association between CP-related SNPs and risk of CKD, and between CKD-related SNPs and risk of CP, using the IVW method. The results are presented in Figure 2. This conventional estimate showed no convincing evidence to support the causal relation between CP and CKD in either of two reversed directions (CP-related SNPs on risk of eGFR, effect = 0.003, 95% confidence interval [CI]: −0.003–0.008, *p* = 0.317; CP-related SNPs on risk of BUN, effect = 0.002, 95% CI: −0.004–0.009, *p* = 0.472; eGFR-related SNPs on risk of CP, effect = −0.333, 95% CI: −3.124–2.459, *p* = 0.815; BUN = −related SNPs on risk of CP, effect = −0.021, 95% CI: −1.447–1.405, *p* = 0.977). For the single genetic instrument, only one of seven SNPs used as genetic instruments in the IVW method demonstrated that CP was causally associated with eGFR (*p* < 0.001 for rs2976950), which changed little of the overall IVW estimate of all CP-related SNPs on the risk of eGFR (*p* = 0.317) (Burgess and Thompson, 2015).

### Causal Association of CP and CKD by Different MR Approaches

**FIGURE 3:**
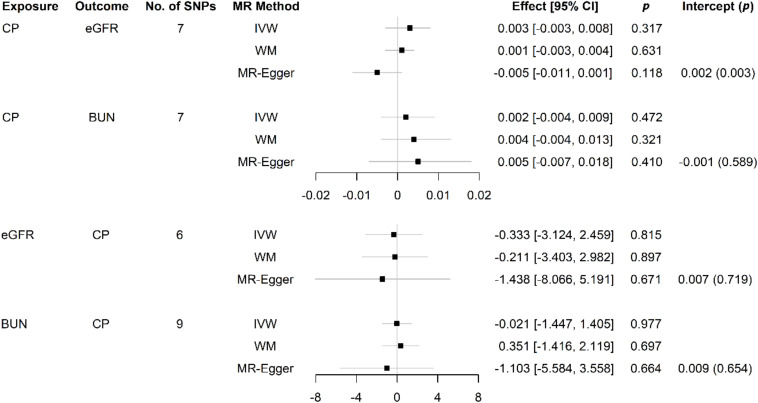
Causal effects between CP and kidney function measures using different MR approaches. SNP: single-nucleotide polymorphism; No.: number; MR: Mendelian randomization; CI: confidence interval; *p*, *p*-value; CP: chronic periodontitis; eGFR: estimated glomerular filtration rate; BUN: blood urea nitrogen; IVW: inverse variance weighted; WM: weighted median; MR–Egger: the Mendelian randomization–Egger method.

The bidirectional MR estimates between CP and CKD by multiple methods are presented in Figure 3. The associations of CP with CKD biomarkers were consistent in the sensitivity analysis that used the WM but not in the MR–Egger method. The intercept test of MR–Egger suggested a potential directional pleiotropy (*p* = 0.003 for CP on eGFR, using all seven SNPs). This was also indicated in the scatter plots in terms of the results from the IVW method (Figure 2).

We assessed the statistical power of this current bidirectional MR study. Based on the variance in CP, eGFR, and BUN according to corresponding seven, six, and nine SNPs, respectively, and the sample sizes of 12,289 cases and 22,326 controls in cohorts from the GLIDE Consortium for CK and 41,395 cases and 4,39,303 controls in cohorts from the CKDGen Consortium for CKD, our study could have over 99% power at an alpha rate of 5% to detect a statistically significant causal effect.

## Discussion

In the present study, we investigated the potential causal roles of CP in the development of CKD and the reverse causal relation of kidney function with the progress of CP, by conducting multiple complementary MR approaches. Using genetic variants as proxies for CP and kidney function measures, including eGFR and BUN, our study did not observe strong evidence to support that genetically predicted CP was associated with decreased eGFR or increased BUN and vice versa.

Previous observational studies based on cross-sectional or case–control design can only describe a connection between CP and CKD because of the absence of chronological sequence (Chen et al., 2006; Kshirsagar et al., 2007; de Souza et al., 2014). In further population-based studies with cohort design, the onset of the exposure can be observed to happen before or after the outcome of interest, but the causal relationship between the traits is yet difficult to be assessed according to the affection by reverse causation or confounding effects (Grubbs et al., 2015, 2016). In the present MR study, we combined the summary statistics of CP and measures of kidney function from large-scale cohorts with European ancestry, to investigate the causal effect on these two traits. To the best of our knowledge, our study performed the first MR analysis on the causal effect between CP and CKD. Also, unlike previous studies based on smaller sample sizes, our two-sample MR study is sufficiently powered to assess a causal relationship between CP and CKD.

Our result is consistent with the conclusion from the newest systematic review and meta-analysis of observational studies on CP and CKD, including seven case–control studies, 38 cross-sectional studies, and two retrospective cohort studies (Zhao et al., 2018). Despite the lack of evidence to support CP as causal factors for CKD from our study, it does not hint that treatment for periodontitis in hemolysis patients with CKD or kidney transplantation patients with ESRD is unnecessary. Improved early treatment and dental care for the prevention of periodontitis could assist in the relief of the overall inflammatory status in the period of hemolysis treatment (Schmalz et al., 2016). Furthermore, for immunosuppressive therapy on patients with kidney transplantation, care of oral and periodontal condition is important for preventing complications and improvement of survival (Kitamura et al., 2019).

The current study has several strengths. First, this study investigated the largest GWAS datasets of CP included in the GLIDE Consortium, analyzing a total of 12,289 clinically diagnosed periodontitis cases and 22,326 controls, and of CKD included in the CKDGen Consortium (41,395 cases and 4,39,303 controls). The participants recruited in these two independent Consortiums are mostly of European descent, which minimizes the influence of population stratification (Burgess and CRP CHD Genetics Collaboration, 2013). Second, our two-sample design estimating the association between the genetic variant exposure and genetic variant outcome was from two independent comparable populations to gain a larger statistical power (Burgess et al., 2015). The bidirectional analysis guarantees the inference of causality between CP and CKD in both directions (Cao et al., 2019). Third, to control the pleiotropic effect from a certain single genetic variable, we used as much as multiple variants robustly associated with exposure variables as genetic instruments for assessing their effect on the outcome variables (Palmer et al., 2012).

Although our study used the newest data available, this study has some potential limitations. MR uses an average risk effect of genetic variants on a specific trait in participants’ lifetime; in such case, it could not answer whether an exposure within a certain period of life has any effect on the risk of an outcome. We used the most recent GWAS of CP and CKD in the population of European ancestry to gain sufficient statistical power to test the potential causal relation between CP and CKD; however, it might be limited to explore a tiny effect between these pairs of traits based on the weak instrument bias. The presented beta and standard error values for all instrumental variables show their effect size; all seven SNPs used as candidate genetic instruments for CP were weakly associated with CP, with a threshold of *p* < 5 × 10^–6^ instead of 5 × 10^–8^. In addition, the functional mechanisms for most of these SNPs related to periodontitis remain unclear. The weak instruments tend to shift the MR estimate toward the null in two-sample MR (Davies et al., 2018), which may therefore result in the uncertain causality between CP and CKD in our study. Future high-quality GWAS are warranted to further examine the potential etiological role of CP in various diseases.

In conclusion, using CP-associated SNPs as genetic instruments retrieved from the GWAS results within large populations with European ancestry, our MR study does not find sufficient evidence to support a causal effect of CP as an exposure on the development of CKD as an outcome. Similarly, in the reverse inference, limited evidence was obtained in support of a causal role of CKD on CP.

## Data Availability Statement

The original contributions presented in the study are included in the article/Supplementary Material, further inquiries can be directed to the corresponding author/s.

## Author Contributions

JY and TC contributed to the protocol development, data collection, and analysis. JY, TC, and YZ drafted the manuscript. MB and XL supervised the method and visualized the results. All authors contributed to manuscript revision, read and approved the submitted version.

## Conflict of Interest

YZ was employed by the company Beijing Lucidus Bioinformation Technologies. The remaining authors declare that the research was conducted in the absence of any commercial or financial relationships that could be construed as a potential conflict of interest.

## Footnotes

1 http://www.phenoscanner.medschl.cam.ac.uk

2 https://shiny.cnsgenomics.com/mRnd/

3 http://gforge.se/packages/
