# Supplementary material for: Causal Inference Between Chronic Periodontitis and Chronic Kidney Disease: A Bidirectional Mendelian Randomization Analysis in a European Population
Source: Front Genet. 2021 Jun 7;12:676136. doi: 10.3389/fgene.2021.676136 (PMC8215666; doi:10.3389/fgene.2021.676136)
Supplement: Supplementary file 1 [file Data_Sheet_1.docx]

**Table S1 |**Basic characteristics of the studies that contributed summary statistics of chronicperiodontitis (CLIDE) and kidney function (CKDGen)

| Characteristic^a^ | GLIDE |
| --- | --- |
| Sample size | 34,615 |
| Age (yr), mean | 59.7 |
| Female percentage | 55.5 |
| CP percentage | 35.5 |
| Characteristic^b^ | CKDGen |
| Sample size | 480,698 |
| Age (yr), median | 50.1 |
| Female percentage | 51.8 |
| eGFR (ml/min per a.73 m^2^), median | 91.4 |
| CKD percentage | 8.6 |

^a^ The basic characteristics of the studies participated in the GLIDE Consortium were calculated as the weighted average of the characteristics of each study reported in previous study(Shungin et al., 2015), with the study specific sample size as the weight.

^b^The basic characteristics of the studies participated in the CKDGen Consortium were calculated by previous study(Yu et al., 2020), using the weighted average of the characteristics of each study reported in the meta-analysis of GWASs on CKD (Wuttke et al., 2019), with the study specific sample size as the weight.

**REFERENCES**

Shungin, D., Cornelis, M. C., Divaris, K., Holtfreter, B., Shaffer, J. R., Yu, Y. H., et al. (2015). Using genetics to test the causal relationship of total adiposity and periodontitis: Mendelian randomization analyses in the Gene-Lifestyle Interactions and Dental Endpoints (GLIDE) Consortium. *Int. J. Epidemiol.* 44, 638-650. doi:10.1093/ije/dyv075

Wuttke, M.Li, Y.Li, M.Sieber, K. B.Feitosa, M. F.Gorski, M., et al. (2019). A catalog of genetic loci associated with kidney function from analyses of a million individuals. *Nat. Genet.* 51, 957-972. doi:10.1038/s41588-019-0407-x

Yu, Z., Coresh, J., Qi, G., Grams, M., Boerwinkle, E., Snieder, H., et al. (2020). A bidirectional Mendelian randomization study supports causal effects of kidney function on blood pressure. *Kidney Int.* 98, 708-716. doi:10.1016/j.kint.2020.04.044

**ACKNOWLEDGEMENTS**

The present study based on summary-level data that have been made publicly available. Summarized statistics from GWAS of chronic periodontitis are available at https://data.bris.ac.uk/data/dataset/2j2rqgzedxlq02oqbb4vmycnc2. The GWAS summary data of chronic kidney disease are available at http://ckdgen.imbi.uni-freiburg.de/.

Acknowledgement of the contributing studies and databases (the GLIDE and the CKDGen projects) that made GWAS summary data available. We gratefully acknowledge the assistance and contributions of Dmitry Shungin, Simon Haworth, KimonDivaris, Cary S. Agler, YoichiroKamatani , MyoungKeun Lee, Kelsey Grinde1, George Hindy, ViiviAlaraudanjoki, Paula Pesonen, Alexander Teumer, BirteHoltfreter, Saori Sakaue, Jun Hirata, Yau-Hua Yu, Paul M. Ridker, Franco Giulianini, Daniel I. Chasman, Patrik K.E. Magnusson, TakeakiSudo, Yukinori Okada, Uwe Völker, Thomas Kocher, VuokkoAnttonen, Marja-LiisaLaitala, MarjuOrho-Melander, Tamar Sofer, John R. Shaffer, Alexandre Vieira, Mary L. Marazita, Michiaki Kubo, Yasushi Furuichi, Kari E. North, Steve Offenbacher, Erik Ingelsson, Paul W. Franks, Nicholas J. Timpson and Ingegerd Johansson, from the GLIDE consortium, and Matthias Wuttke, Yong Li, Man Li, Karsten B. Sieber, Mary F. Feitosa, Mathias Gorski, Adrienne Tin, Lihua Wang, Audrey Y. Chu, Anselm Hoppmann, Holger Kirsten, AyushGiri, Jin-Fang Chai, GardarSveinbjornsson, Bamidele O. Tayo, Teresa Nutile, Christian Fuchsberger, Jonathan Marten, Massimiliano Cocca, Sahar Ghasemi, Yizhe Xu, Katrin Horn, DamiaNoce, Peter J. van der Most, SanazSedaghat, Zhi Yu, Masato Akiyama, Saima Afaq, Tarunveer S. Ahluwalia, Peter Almgren, Najaf Amin, Johan Ärnlöv, Stephan J. L. Bakker, Nisha Bansal, Daniela Baptista, Sven Bergmann, Mary L. Biggs, Ginevra Biino, Michael Boehnke, Eric Boerwinkle, Mathilde Boissel, Erwin P. Bottinger, Thibaud S. Boutin, Hermann Brenner, Marco Brumat, Ralph Burkhardt, Adam S. Butterworth, Eric Campana, Archie Campbell, Harry Campbell, MickaëlCanouil, Robert J. Carroll, Eulalia Catamo, John C. Chambers, Miao-Ling Chee, Miao-Li Chee, Xu Chen, Ching-Yu Cheng, Yurong Cheng, Kaare Christensen, Renata Cifkova, Marina Ciullo, Maria Pina Concas, James P. Cook, Josef Coresh, Tanguy Corre, Cinzia Felicita Sala, Daniele Cusi, John Danesh, E. Warwick Daw, Martin H. de Borst, Alessandro De Grandi, Renée de Mutsert, Aiko P. J. de Vries, Frauke Degenhardt, Graciela Delgado, AyseDemirkan, Emanuele Di Angelantonio, Katalin Dittrich, Jasmin Divers, Rajkumar Dorajoo, Kai-Uwe Eckardt, Georg Ehret, Paul Elliott, KarlhansEndlich, Michele K. Evans, Janine F. Felix, Valencia Hui Xian Foo, Oscar H. Franco, Andre Franke, Barry I. Freedman, Sandra Freitag-Wolf, Yechiel Friedlander, Philippe Froguel, Ron T. Gansevoort, He Gao, Paolo Gasparini, J. Michael Gaziano, Vilmantas Giedraitis, Christian Gieger, GiorgiaGirotto, Franco Giulianini, Martin Gögele, Scott D. Gordon, Daniel F. Gudbjartsson, VilmundurGudnason, Toomas Haller, Pavel Hamet, Tamara B. Harris, Catharina A. Hartman, Caroline Hayward, Jacklyn N. Hellwege, Chew-Kiat Heng, Andrew A. Hicks, Edith Hofer, Wei Huang, Nina Hutri-Kähönen, Shih-Jen Hwang, M. Arfan Ikram, Olafur S. Indridason, Erik Ingelsson, Marcus Ising, Vincent W. V. Jaddoe, Johanna Jakobsdottir, Jost B. Jonas, Peter K. Joshi, Navya Shilpa Josyula, Bettina Jung, Mika Kähönen, YoichiroKamatani, Candace M. Kammerer, Masahiro Kanai, Mika Kastarinen, Shona M. Kerr, Chiea-Chuen Khor, Wieland Kiess, Marcus E. Kleber, Wolfgang Koenig, Jaspal S. Kooner, Antje Körner, Peter Kovacs, Aldi T. Kraja, Alena Krajcoviechova, Holly Kramer, Bernhard K. Krämer, Florian Kronenberg, Michiaki Kubo, Brigitte Kühnel, MikkoKuokkanen, Johanna Kuusisto, Martina La Bianca, Markku Laakso, Leslie A. Lange, Carl D. Langefeld, Jeannette Jen-Mai Lee, Benjamin Lehne, TerhoLehtimäki, Wolfgang Lieb, Lifelines Cohort Study, Su-Chi Lim, Lars Lind, Cecilia M. Lindgren, Jun Liu, Jianjun Liu, Markus Loeffler, Ruth J. F. Loos, Susanne Lucae, Mary Ann Lukas, Leo-PekkaLyytikäinen, ReedikMägi, Patrik K. E. Magnusson, Anubha Mahajan, Nicholas G. Martin, Jade Martins, Winfried März, Deborah Mascalzoni, Koichi Matsuda, Christa Meisinger, Thomas Meitinger, Olle Melander, Andres Metspalu, Evgenia K. Mikaelsdottir, Yuri Milaneschi, KozetaMiliku, Pashupati P. Mishra, V. A. Million Veteran Program, Karen L. Mohlke, Nina Mononen, Grant W. Montgomery, Dennis O. Mook-Kanamori, Josyf C. Mychaleckyj, Girish N. Nadkarni, Mike A. Nalls, Matthias Nauck, KjellNikus, Boting Ning, Ilja M. Nolte, Raymond Noordam, Jeffrey O’Connell, Michelle L. O’Donoghue, IsleifurOlafsson, Albertine J. Oldehinkel, MarjuOrho-Melander, Willem H. Ouwehand, Sandosh Padmanabhan, Nicholette D. Palmer, RunolfurPalsson, Brenda W. J. H. Penninx, Thomas Perls, Markus Perola, Mario Pirastu, Nicola Pirastu, Giorgio Pistis, Anna I. Podgornaia, OzrenPolasek, Belen Ponte, David J. Porteous, Tanja Poulain, Peter P. Pramstaller, Michael H. Preuss, Bram P. Prins, Michael A. Province, Ton J. Rabelink, Laura M. Raffield, Olli T. Raitakari, Dermot F. Reilly, Rainer Rettig, Myriam Rheinberger, Kenneth M. Rice, Paul M. Ridker, Fernando Rivadeneira, Federica Rizzi, David J. Roberts, Antonietta Robino, Peter Rossing, Igor Rudan, Rico Rueedi, Daniela Ruggiero, Kathleen A. Ryan, Yasaman Saba, CharumathiSabanayagam, VeikkoSalomaa, Erika Salvi, Kai-Uwe Saum, Helena Schmidt, Reinhold Schmidt, Ben Schöttker, Christina-Alexandra Schulz, Nicole Schupf, Christian M. Shaffer, Yuan Shi, Albert V. Smith, Blair H. Smith, Nicole Soranzo, Cassandra N. Spracklen, Konstantin Strauch, Heather M. Stringham, Michael Stumvoll, Per O. Svensson, Silke Szymczak, E-Shyong Tai, Salman M. Tajuddin, Nicholas Y. Q. Tan, Kent D. Taylor, Andrej Teren, Yih-Chung Tham, Joachim Thiery, Chris H. L. Thio, Hauke Thomsen, GudmarThorleifsson, Daniela Toniolo, AnkeTönjes, Johanne Tremblay, IoannaTzoulaki, André G. Uitterlinden, Simona Vaccargiu, Rob M. van Dam, Pim van der Harst, Cornelia M. van Duijn, Digna R. Velez Edward, Niek Verweij, Suzanne Vogelezang, Uwe Völker, Peter Vollenweider, Gerard Waeber, Melanie Waldenberger, Lars Wallentin, Ya Xing Wang, Chaolong Wang, Dawn M. Waterworth, Wen Bin Wei, Harvey White, John B. Whitfield, Sarah H. Wild, James F. Wilson, Mary K. Wojczynski, Charlene Wong, Tien-Yin Wong, Liang Xu, Qiong Yang, Masayuki Yasuda, Laura M. Yerges-Armstrong, Weihua Zhang, Alan B. Zonderman, Jerome I. Rotter, Murielle Bochud, Bruce M. Psaty, Veronique Vitart, James G. Wilson, Abbas Dehghan, Afshin Parsa, Daniel I. Chasman, Kevin Ho, Andrew P. Morris, Olivier Devuyst, ShreeramAkilesh, Sarah A. Pendergrass, Xueling Sim, Carsten A. Böger, Yukinori Okada, Todd L. Edwards, Harold Snieder, Kari Stefansson, Adriana M. Hung, Iris M. Heid, Markus Scholz, Alexander Teumer, Anna Köttgen and Cristian Pattaro from the CKDGen consortium.
